# Pediatric Rectal Prolapse: A Case Report

**DOI:** 10.7759/cureus.76432

**Published:** 2024-12-26

**Authors:** Melissa A Mallary, Bansri S Patel, Victoria M Arredondo, Natasha Fein-Davis

**Affiliations:** 1 Emergency Medicine, A.T. Still University - School of Osteopathic Medicine in Arizona, Mesa, USA; 2 Pediatrics, A.T. Still University - School of Osteopathic Medicine in Arizona, Mesa, USA

**Keywords:** colonoscopy, gastroenterology, general surgery, pediatrics, rectal mass, rectal prolapse

## Abstract

In the absence of preexisting conditions, rectal prolapse is rarely seen in children older than four years old. This case report presents a peculiar instance involving a previously healthy five-year-old female who presented to the clinic with her parents due to a three-day history of constipation, hard stools, and painful defecation. Physical examination revealed a rectal prolapse. Given the infrequency of rectal prolapse without underlying conditions in this age group, the patient was referred to a tertiary emergency department at the children’s hospital to be evaluated by pediatric gastroenterology. Most rectal prolapse cases in children resolve without intervention; however, in children over the age of four, it may require surgery due to the likelihood of an underlying neurological or musculoskeletal condition. This case emphasizes the value of a thorough physical examination to detect the finding and stresses the importance of ruling out a more serious etiology.

## Introduction

Rectal prolapse is a condition marked by the protrusion of some or all of the rectal mucosa that has passed through the external anal sphincter [1]. Without predisposing factors, rectal prolapse is an unusual finding in children, although if present, it generally presents between infancy and four years of age [1]. The highest incidence of rectal prolapse has been reported within the first year of life [2]. Some sources report that weak pelvic floor musculature predisposes young children to prolapse [2]. It is common for the condition to be first noticed by parents or guardians who notice the abnormality of a dark red mass extruding from the rectum, which initiates the concern to seek medical attention [2]. The mass is usually seen during episodes of straining and occasionally encompasses mucus or blood [2].

In pediatric cases, rectal prolapse most likely indicates an underlying condition; therefore, a comprehensive diagnostic evaluation should be performed [1]. In the United States, rectal prolapse is thought to be linked with cystic fibrosis (CF). However, it is mainly associated with stool abnormalities such as acute diarrhea and chronic constipation or with neurologic and anatomical defects [1,3]. In an otherwise healthy child with suspected idiopathic rectal prolapse, CF needs to be ruled out [1,3,4]. Predisposing factors in developing nations include acute diarrheal disease and, in contrast, intestinal parasitic infestations, often as a result of malnutrition [1,4].

There are two principal types of rectal prolapse: Type I, also known as false providential, which involves partial or mucosal prolapse and is generally less than 2 cm in length, and Type II, also known as true providential, which is defined by full-thickness protrusion of the rectal wall with the presence of concentric folds within the prolapsed mucosa [1].

The pathogenesis of rectal prolapse is recognized to be due to the circumferential intussusception of the upper rectum and rectosigmoid colon [1]. Anatomical factors make certain patients more susceptible to rectal prolapse [1]. Factors include a rectum with a vertical orientation, excessive fat in the coccygeal region, inadequate levator ani muscular support, and hypermobility of the sigmoid colon [1]. In children under four years of age, rectal prolapse is generally self-limiting and may resolve spontaneously; however, if external exposure of the mass is prolonged, it can result in complications such as ulceration, venous obstruction, and thrombosis [1,4]. If a child older than four years of age presents with rectal prolapse, there is a greater chance of an underlying neurological or musculoskeletal defect that will not likely respond to conservative management; moreover, early referral for surgical intervention is recommended to effectively focus on correcting the underlying issue [1,4].

## Case presentation

A five-year-old female without a chronic history of constipation was brought into our pediatric office by her parents for evaluation of acute onset of constipation and rectal bleeding. The parents reported that the patient had been experiencing intermittent constipation and rectal pain with defecation for the past two to three days. The patient’s symptoms included hard stools, straining with defecation, and rectal pain. The parents stated that the patient’s symptoms had worsened, which prompted them to seek medical care. The patient’s associated symptoms included abdominal pain, abdominal cramping, and bright red blood on the toilet paper. The patient denied experiencing fever, nausea, vomiting, heartburn, mucus in stool, black or tarry stools, weakness, urinary symptoms, or rash. There was no pertinent past medical, surgical, family, or social history. The patient had no known drug allergies, per her parents.

The patient was active, alert, not in acute distress, and attentive on the physical exam. Examination demonstrated pupils were equal, round, and reactive to light, and the nose was patent without polyps. No oral ulcers were noted. Heart sounds were normal, with regular rate and rhythm and no murmurs. The lungs were clear to auscultation bilaterally without wheezing, rales, or rhonchi. The patient reported generalized tenderness on abdominal palpation, but no masses or hepatosplenomegaly were palpated. The neurologic exam showed normal strength, tone, and reflexes of the extremities. During genitourinary evaluation, blood was found to be present in the patient’s underwear, and a large rectal prolapse was present. The patient’s skin was dry, without rashes or cyanosis.

The patient was diagnosed with rectal prolapse secondary to constipation based on clinical presentation and physical examination. Due to the severity of the patient’s symptoms, pediatric gastroenterology was consulted and advised that the patient be evaluated at the emergency department, and gastroenterology would consult upon arrival. The parents were informed of this recommendation and agreed to transport the child to the emergency department. However, per the emergency room physician’s note, the patient’s rectal prolapse had spontaneously resolved before arrival without signs of active bleeding, and the patient was discharged home with a prescription for MiraLAX to assist with constipation. Unfortunately, no records were sent to our office following this episode, and the only phone number available to us was no longer in service. Therefore, it is unclear whether the patient followed up with gastroenterology or if her symptoms remained resolved.

## Discussion

Rectal prolapse seldom occurs in children who do not have underlying predisposing conditions [4,5]. When found in children, it is usually self-limiting in younger kids less than four years of age and more often found in young boys than girls [4,5]. A Type I rectal prolapse, also known as false procidentia, involves protrusion of the mucosa only and is usually less than 2 cm long [5,6]. A Type II rectal prolapse, also known as true procidentia, involves full-thickness extrusion of the rectal wall and is characterized by concentric folds in prolapsed mucosa [5,6]. Type II can be further subdivided into three divisions. The first degree includes the mucocutaneous junction, and the distance of protrusion from the anal verge is more than 5 cm [6]. The second degree does not involve the mucocutaneous junction, and the distance of protrusion from the anal verge is 2 to 5 cm [6]. The third degree is internally concealed or occult and does not pass through the anal verge [6].

The pelvic floor anatomy in children varies slightly from that of fully grown adults [1]. For instance, the rectum is vertical along the surface of the sacrum and coccyx and is lower than other pelvic organs [1]. The sigmoid is very mobile, and the levator ani muscles offer very little support [1]. Redundant tissue is only attached loosely to the underlying muscularis [1]. Additionally, Houston valves, which are prominent mucosal folds providing structural support to the rectum, are absent in 75% of infants under one year old [1].

For children over four years old, where rectal prolapse may not be self-limiting, it is crucial to consider predisposing conditions [4,6]. Any increase in intra-abdominal pressure can result in prolapse [4,6,7]. Although current recommendations do not include abdominal imaging, if the clinical picture fits, imaging may be indicated to rule out intra-abdominal tumors as a cause of increased abdominal pressure leading to rectal prolapse [7]. This includes but is not limited to chronic constipation, coughing, toilet training, vomiting, and straining with urination [3,6,7]. Additionally, children with CF and children who were exposed to diarrheal diseases caused by Shigella, parasites, or pinworms can all have a higher chance of experiencing rectal prolapse [1,7]. However, since universal screening was implemented in the United States, the incidence of rectal prolapse in patients with CF has diminished significantly due to early diagnosis and treatment of CF [3,7,8]. Malnutrition is another predisposing condition to rectal prolapse because it causes hypoproteinemia, leading to mucosal edema, and also reduces the ischiorectal fat pad. Reducing the ischiorectal fat pad decreases perirectal support [3,7]. Weakening of the pelvic floor because of neurological or previous surgical interventions should also be taken into consideration when working up the cause of rectal prolapse [3,5].

To diagnose rectal prolapse, a thorough history and physical exam must be completed [1,5,6]. On a physical exam, there may be a painless, dark mass at the anal verge without mucus present [1,5,6]. Examining the child in a squatting position and performing palpation is essential [1,5]. If an occult or internal rectal prolapse is suspected, a sigmoidoscopy generally reveals a red and granular distal rectum [1,5,7]. In the differential diagnosis, it is also essential to consider an ileocecal intussusception, prolapsing rectal polyp, and rectal hemorrhoids [1,5,6].

In children less than four years old, rectal prolapse is generally self-limiting. In older pediatric patients, management is typically conservative and focused on diagnosing and treating predisposing conditions mentioned above [1,4,5,6]. Manual reduction should be attempted if spontaneous reduction does not occur [3,5]. Between the two types of rectal prolapse, Type II rectal prolapse is rarer and often requires surgical intervention [9,10]. The basis of surgical repair involves restoring the normal posterior curve of the rectum while also repairing any anterior pelvic floor defects in a procedure called a rectopexy [10]. There have been multiple case reports utilizing a rectopexy procedure for failed manual reduction of rectal prolapse without recurrence [10,11]. Generally speaking, the prognosis is usually good depending on the underlying condition, but it is important to teach parents how to reduce it in case of recurrence [5,6,9,10]. Surgical correction may be needed if prolapse is recurrent [3,9,10].

## Conclusions

A rectal prolapse is the partial or complete extrusion of the rectal mucosa through the external sphincter due to an underlying gastrointestinal condition. Rectal prolapse is uncommon in children older than four, especially without an underlying predisposing condition. The most likely cause of rectal prolapse in children older than four years of age is constipation. Constipation causes increased intra-abdominal pressure with straining, and the increased pressure may overpower the strength of the external anal sphincter, resulting in protrusion of the rectal mucosa. The purpose of this case report is to highlight that despite rectal prolapse being uncommon in children over the age of four, it is still essential to perform an anal examination on a patient complaining of constipation, rectal discomfort, or rectal bleeding to assess if a prolapse has occurred. In children over the age of four, treatment for rectal prolapse may not respond to conservative management and requires surgical repair to treat the prolapse and prevent recurrence.
